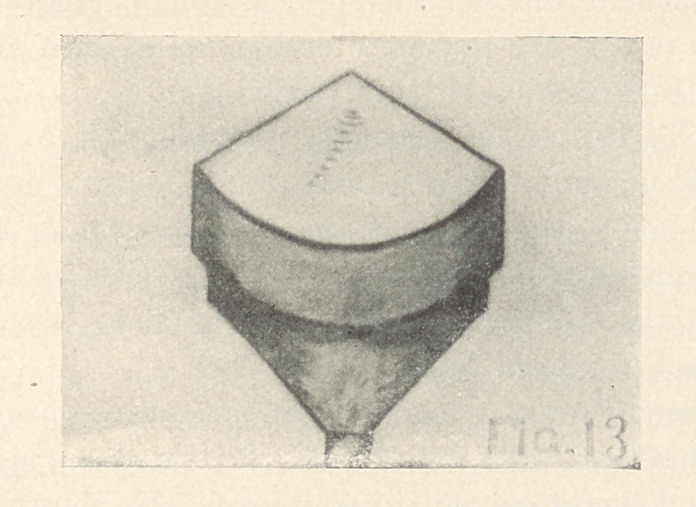# The Rationale of the Porcelain Inlay

**Published:** 1905-08

**Authors:** J. Q. Byram

**Affiliations:** Indianapolis, Ind.


### International Dental Journal.

VOL. XXVI. AUGUST, 1905. No. 8.

### Original Communications.[1]

### THE RATIONALE OF THE PORCELAIN INLAY.[2]

BY DR. J. Q. BYRAM, INDIANAPOLIS, IND.

I WISH to express my appreciation of the honor conferred upon me by your society in inviting me to appear as your essayist this evening. I assure you that I feel my incompetency to read a paper on a subject which is not only familiar to you, but one on which several of your members are authority.

It seems quite impossible to add anything of value to the discussion of porcelain inlays; so much has been written on this subject within the last four years. But a repetition of the old enables one to become more familiar with a problem. That there is a growing interest in porcelain as a filling-material is indicated by the number of dentists who are becoming skilful in this branch of dental art. Yet the majority of dentists know little about porcelain, and many believe that the insertion of porcelain inlays is a fad. It is probable that many of the enthusiasts have gone to extremes, and that many articles have been written which deal with the ideal rather than the practical, and that those writers

[1] The editor and publishers are not responsible for the views of authors of papers published in this department, nor for any claim to novelty, or otherwise, that may be made by them. No papers will be received for this department that have appeared in any other journal published in the country.

[2] Read before the Academy of Stomatology, Philadelphia, Pa., March 28, 1905.

who have been so fanciful should be classed as visionaries and idealists. But the idealist by his enthusiasm has been an educator to a keener appreciation of artistic dentistry. We need more hobbyists on the subject of porcelain; for had it not been for the enthusiasts, its virtues would not be admitted to the extent that they are to-day.

One of the elements of true dental art consists in disguising the artificiality of the material by which lost tissue or organs are replaced. If the enthusiastic exponents of porcelain inlays, in their desire to conceal their art, have gone beyond reasonable limits in the use of inlays, this enthusiasm has caused many dentists to develop their skill and æsthetic sense to a higher degree, thus enabling them to practise dentistry in a more artistic manner.

Porcelain is now recognized as a meritorious filling-material, and its adoption will come of necessity, if not from choice. It will be but a short time until the dentist who fails to master the technique of constructing porcelain inlays will be relegated to the rear ranks of the profession; for porcelain is the material which should be used in filling most conspicuous cavities.

It is true that, at the present time, the extent of the use of porcelain as a filling-material is dependent on the dentist. Many dentists will be required to develop their æsthetic sense before it can be universally used. Much has been said about educating the public to a higher appreciation of artistic dentistry, but until more dentists are educated along this line we can hardly expect to make much progress in the education of the laity. Paradoxical as it may seem, however, the laity is assisting in educating the profession.

Many dentists are discouraging the use of porcelain, because they are not familiar with its good qualities. Some may have seen a few failures, and because of ignorance of its properties are condemning it. Others, through inexperience and lack of knowledge of the properties of porcelain, have made failures. So they are condemning it.

The construction of porcelain inlays involves four sets of manipulative principles:

1. The preparation of the cavity.

2. The construction of the matrix.

3. The selection, the application, and the fusing of the porcelain.

4. The finishing and setting of the inlay.

Each of these steps involves a series of mechanical principles, and if they are not executed according to these principles, an imperfect filling will be the result. The failure of many inlays may be attributed to lack of detail, for too many beginners attempt the construction of inlays for the mouth before they have mastered the technique.

There is a difference of opinion regarding the preparation of cavities for inlays. Some insist that inlays can be retained in cavities without any retentive form, and that their retention is wholly dependent upon close adaptation and the adhesive properties of the cement. Others believe that cavities should be given as nearly a self-retentive form as it is possible to make, in order to resist the tendency of dislodgement of the filling under stress. They also believe that the cement tends to stay inlays rather than retain them. Those who follow the teachings of the latter will have fewer inlays to reconstruct or reset. Cavities for inlays should be prepared with the same carefulness that should be used in the preparation of cavities for other fillings. The mechanical principles by which inlays are retained must be observed with absolute accuracy, and all cavities should be given a retentive form to resist stress. The cavity must be sufficiently deep to give ample thickness of porcelain, which will insure structural strength and avoid defects in the construction of the inlay. The cavity margins should be formed to avoid unsupported enamel-rods, they should be smooth and in curves or straight lines, avoiding angles whenever possible. There should be no bevel of the enamel margins to give frail edges of porcelain. Two requirements for the proper construction of matrices are accessibility and working space. It is essential that teeth with proximal cavities be separated to insure the proper withdrawal of the matrix and the insertion of the inlay.

### THE PREPARATION OF CAVITIES.

Dr. C. N. Thompson [1] suggests that the walls of a labial or a buccal cavity should be at right angles with the curves of the surface (Fig. 1). This causes them to converge slightly toward the pulpal wall. The pulpal wall should be flat and its junction with the other walls should be in curves instead of angles. The cavity

---

[1] Dental Digest, May, 1904.

should be deep enough to afford the best possible anchorage and to prevent the cement's changing the color of the inlay.

Fig. 1

In simple proximal cavities involving both labial and lingual surfaces the gingival wall should form a right angle with the pulpal wall. The incisal wall should form a right angle with the curve of the proximal surface (Fig. 2, A). These walls should

Fig. 2

extend far enough to prevent frail margins of porcelain, but care should be exercised to guard against undue weakness of the incisal angle. The labial and lingual walls should extend far enough to reach sound enamel, and these margins should unite with the gingival and the incisal margins in curves. The cavity should have no undercuts or sharp angles between these walls. The lingual wall may extend farther than the labial, and a step may be cut in the middle and gingival thirds (Fig. 2, B), to insure additional retentive resistance.

Because of the variation in proximo-incisal cavities it is almost impossible to outline a conventional form of cavity preparation. Proximo-incisal cavities may be divided into (1) cavities involving but a small portion of the labial and the lingual surfaces, and (2) extensive cavities requiring a step for additional retentive resistance.

THE PREPARATION OF SMALL PROXIMO-INCISAL CAVITIES.

The gingival wall should be flat, but its margins should be concave mesio-distally (Fig. 3, A). The incisal enamel margins

Fig. 3

should be extended to avoid frail enamel. Cut a triangular cavity, extending from the gingival to the incisal wall, and having parallel walls, as deep as the pulp will permit (Fig. 3, B). A concavity should be made in the dentine of the incisal wall, after the inlay has been completed, and a groove in the ridge on the inlay (Fig. 3, C), giving it something of a mortise form.

PROXIMO-INCISAL CAVITIES WITH STEPS.

Since the necessity for retentive resistance increases in ratio to the increased width of the inlay, a step should be made in large proximo-incisal cavities to resist the tipping stress. The gingival wall of the cavity should be flat, with its margins concave mesio-distally. The labial and lingual walls should be extended parallel until sound enamel, supported by dentine, is reached. Then a shallow cavity should be cut between the labial and lingual walls (Fig. 4, A). A step should be prepared, involving enough toothstructure to give a mass of porcelain large enough to withstand the force of mastication.

PREPARATION OF THE STEP.

The step should be cut across the incisal edge, extending from one and one-half to three millimetres; it should be from one to

Fig. 4

two millimetres deep on the labial surface, and from one and one-half to three millimetres deep on the lingual. The labial and lingual margins should both form right angles with the gingivo-incisal curves of these surfaces (Fig. 4, B), and they should form

Fig. 5

slight reverse curves with the axial margins of the cavity (Fig. 4, C). A shallow cavity may be cut between the labial and the lingual plates when they are approximately the same length (Fig. 5, A). The lingual plate should be cut almost one-half the thickness of the incisal end if it is extended farther gingivally than the labial plate (Fig. 4, B). It should be so formed that its gingival wall will be at right angles with the concavity of the lingual surface.

A step may also be formed involving only the lingual plate in those teeth with thick incisal edges (Fig. 6, A). It should involve at least one-half the thickness of the incisal edge, extending from one and one-half to three millimetres gingivally. The gingival wall should form a right angle with the concavity of the lingual surface (Fig. 6, B). A slight groove may extend along the labioproximal angle (Fig. 6, C). Dr. J. E. Nyman[1] suggests a method of preparing proximo-incisal cavities by cutting a series of reverse curves as illustrated in Fig. 7. He says, "This irregular outline results in a less conspicuous line of demarcation between the

---

[1] Dental Summary, January, 1905.

porcelain and the tooth enamel than if the margin were cut in a straight line and obtains the essential right-angle margins."

CAVITIES INVOLVING THE INCISAL EDGE.

Simple cavities are usually caused by malformation. The mesial and distal walls of these should extend gingivally from two to three millimetres, and should slightly diverge toward the incisal edge (Fig. 8, A). They should be slightly grooved between

Fig. 8.

their plates of enamel to resist the lingual stress (Fig. 8, B). The seat should slope toward the centre from the labial and lingual margins (Fig. 8, C).

Cavities involving the entire incisal edge are very rare. The inlay must be retained by pins (Fig. 9) or by a step cut on the lingual surface (Fig. 4, B).

Fig. 9

Porcelain is not the safest material for filling small cavities in bicuspids and molars. Their occlusal surfaces present a series of inclined planes, which, when restored with a friable material, lacking in edge strength, cause imperfect fillings in a short time by the breaking down of frail margins. Porcelain should not be used in this class of cavities unless they are in the line of vision. Gold inlays or fillings may be inserted which are just as compatible with the tooth-structure as porcelain.

PREPARATION OF CAVITIES FOR BICUSPIDS AND MOLARS.

The gingival wall should form a right angle with the pulpal wall. The buccal and lingual walls should diverge toward the proximal and occlusal margins. They should be grooved in the dentine occluso-gingivally to form mortices (Fig. 10, A). When the buccal and lingual surfaces are involved, they should extend far enough to prevent frail margins of porcelain (Fig. 10, B). If a step is required, it should extend wide and deep enough to insure strength to the porcelain.

In extensive cases the buccal and lingual cusps should be involved and the cavity margins extended on the buccal surfaces, obliterating to a large degree frail margins of porcelain on the occlusal surface (Fig. 11).

For pulpless molars, where the entire occlusal surface is involved, the pulp-chamber is so prepared that its axial walls are

Fig. 12

slightly divergent (Fig. 12, A). The occlusal margins are bevelled to form slight obtuse angles with the axial walls.

CONSTRUCTING THE MATRIX.

There are three general methods of constructing matrices:

1. Swaging the foil over a negative or an impression of the cavity.

2. Swaging into a positive of the cavity.

3. Burnishing directly into the cavity.

A few dentists claim that they can construct more accurately fitting inlays by swaging matrices over an impression of the cavity. While this method may be good for the construction of matrices for all classes of cavities, and its advocates may construct inlays which accurately fit the cavities before they are cemented in position, my experience with this method has not been entirely satisfactory. Experiments show that the thinnest film to which a layer of cement can be squeezed, under pressure equivalent to that applied in setting an inlay, is about one-thousandth of an inch. So the inlay, when set with cement, will not absolutely fit the cavity.

The method of swaging into a positive of the cavity has some advocates, but it hardly seems possible that a positive of the cavity can be obtained that will produce the cavity margins with that degree of accuracy required in inlay work. I do not regard it, therefore, as a method by which the best results may be obtained.

The method of burnishing the foil directly into the cavity has more advocates than either of the others. And many who have tried the swaging process have returned to this method. A combination of the swaging and burnishing methods can be used successfully in the construction of matrices for large cavities. The technique of constructing a matrix is as follows: Take an impression of the cavity preferably in cement. Then construct a positive of the cavity by mixing a mass of cement and forcing it over the impression, or by casting with a low-fusing metal. After the positive has been separated, it should be invested in the ring of a swaging device. Coat the positive of the cavity thus obtained with soap-stone. Place a piece of foil over it and swage with a velum rubber plunger, so constructed that it presses the foil from the centre of the cavity to the margins (Fig. 13), remove the

Fig. 13

matrix, and paint its margins with a thin solution of shellac. Fill the floor of the matrix with porcelain and fuse to a high biscuit. Insert the matrix into the cavity, and burnish the foil to the margins. This method is especially good in large cavities, and it has the advantage of conforming the foil to the cavity in such a manner that the burnishing is made quite easy.

SELECTION, APPLICATION, AND FUSING OF PORCELAIN.

A study of the colors of the teeth should be made prior to the selection of the porcelain. While the pigments of the tooth determine its color to a large degree, the thickness of the dentine and the enamel, and the density of these tissues, with their power to absorb and refract light, are factors. The colors of most teeth containing no foreign pigments are brown, yellow, gray with yellowish or bluish hue, and blue. Normal dentine is some shade of yellow, while the enamel seems to be a variety of colors.

The variation of color so often noticeable when an inlay is cemented in place is quite perplexing. The inlay may be a good match when the incidence of light rays is at an angle that will permit of their transmission. But when the angle of incidence is changed, some portion of the inlay may appear a different color. The cement is also a factor in the color problem. If it were transparent, it would not prevent the passage of light rays through the tooth. But when light is incident upon an opaque cement, it is excluded from the dentine, and the color of an opaque body is due to the kind of light it reflects, the remaining colors being wholly or partly absorbed on the surface.

A "shadow" is formed when an inlay is cemented into a cavity with a cement which dimly reflects and partially absorbs the transmitted rays if the incidence of light is at such an angle that unabsorbed rays cannot be transmitted.

Dr. W. T. Reeves, of Chicago, was the first to suggest that inlays should be built in layers, and claims for this method a more translucent inlay, avoidance of the "shadow," and a prevention of the cement's reflecting from beneath. This method of constructing inlays will assist in overcoming these obstacles, but it does not wholly remove them.

Mr. Robert Brewster has produced a white opaque porcelain for lining matrices. He says for it: "It is of a bright reflecting character and dense enough to counteract the absorbing effect of the cement. That portion of the inlay to be in contact with the cement is made of this lining. The matrix is then filled with the regular porcelain. When the light strikes the inlay the transmitted rays are reflected through the inlay, thus preventing the absorption of the light rays by the cement."

In the selection of colors for an inlay, note the variation of color of the natural tooth. There are usually three or more colors or shades of colors in a tooth. The foundation of the inlay, representing the dentine, should be yellow (presuming the tooth is vital), while the overlying colors should approach the color of the enamel in their respective positions. The foundation body should be applied and contoured to replace the dentine. It should be fused, provided it is of a higher fusing porcelain than that used to replace the enamel. Care should be exercised to prevent porcelains of different colors from mixing when applying them to their respective positions. If the colors are applied separately and biscuited, then a uniform color be applied over the entire mass and properly fused, the colors will be true and the fused porcelain will have a more natural appearance.

The fusing of the porcelain is as exacting as any step in the construction of the inlay. It is difficult to fuse porcelain at a definite degree of heat without some means of measuring it, and many otherwise good inlays are spoiled in the furnace. Variations in the heat of the muffle will cause variations in the shade and strength of the porcelain. Underfused porcelain is of a darker hue and less translucent, while overfused porcelain is lighter. In either case the porcelain is more porous because of the imperfect rearrangement of the molecules. Overfused porcelain, the result of repeated or prolonged contact with its maximum heat, produces an ebulition of the double silicates, and this causes a blistering of the masses and a change of color.

Since the introduction of the dental pyrometer, the fusing of porcelain has been simplified and positive results can be obtained. To Dr. W. A. Price, of Cleveland, Ohio, belongs the credit of placing the first dental pyrometer on the market. The pyrometer designed by Dr. Price depends upon the principle of a thermopile. When certain metals are subjected to heat, an electrical current is generated, the quantity of which depends upon the temperature to which these metals are subjected. Every increase in the temperature causes a corresponding increase in the quantity of current given off by these metals. A small pellet of the metal rhodium is welded to two platinum wires, and one or more of these miniature thermopiles are introduced in the rear of the muffle. When they are heated, a feeble current is generated. As the temperature increases, the quantity of current in a like manner increases, and this is measured by means of a delicate millameter. Hence the amount of current generated is accurately recorded by this delicate instrument.

The pyrometer designed by Mr. N. K. Garhart, of Indianapolis, Ind., depends upon an entirely different principle. He interposes in the current of a low voltage circuit a "Nernst Glower," and measures the quantity of current passing through the "glower" by means of a delicate millameter. The "Nernst Glower" is a peculiar variety of porcelain which is a non-conductor when cold but a conductor when heated. The conductivity of the "glower" is in ratio to the increased temperature of the muffle. The amount of current required for the "glower" is so small in quantity and slight in pressure that the "glower" seems to undergo no change from repeated use. The current is obtained from the street circuit on the shunt plan, and owing to the minute amount required for this purpose, slight fluctuations of the street supply current do not impair the accuracy of the instrument.

Very little finishing of the inlay is required if proper care is exercised in applying the porcelain. All margins of porcelain overhanging the enamel margins should be removed with cuttlefish disks. In order to insure better adhesion of the porcelain to the cement, that portion of the inlay which is to be inserted into the cavity should be grooved and present a roughened surface. The roughening of the surface is best accomplished by embedding the margins of the inlay in wax, then painting the surface with hydrofluoric acid until it becomes sufficiently roughened. The acid should then be neutralized and the inlay thoroughly cleansed and dried.

The ideal cement for setting inlays should be transparent and very adhesive. But since we are compelled to use an opaque cement, the white is probably the best color to use in setting large proximal inlays. A variety of colors can be obtained from the white cement by the use of pigments without any apparent detrimental effect on the cement. The pigments are useful in coloring the cement the color of the enamel for labial cavities, for the incidence of light on inlays, in cavities on the labial surfaces, is always in such direction that the rays are feebly reflected and partially absorbed by the cement.

In order to obtain the greatest adhesiveness, the cement powder and liquid should be mixed in definite proportions, and they should be thoroughly spatulated. Three grains of powder and two drops of liquid seem to give the proper consistency for Ames's hydraulic cement, while four grains of powder and two drops of liquid seem to give the best results for the Harvard slow-setting cement.

The cavity should be washed with chloroform and dried. A thin film of cement should be placed over the walls and margins of the cavity. The inlay is then forced into position with soft wood or semi-vulcanized rubber; it should be retained in position with wedges or floss tied around it, to allow the cement to crystallize under pressure. After the cement has thoroughly crystallized the surplus is removed and the margins of the inlay are polished with cuttle-fish disks and Arkansas stones.

The fact that inlays are held in position with cement has caused many dentists to doubt the durability of these fillings. The permanency of the cement depends upon the close adaptation of the inlay to the cavity margins, for clinical experience has demonstrated that the dissolution of a thin film of cement between the cavity and the inlay margins is only for a slight depth, leaving the margins sealed to prevent recurrence of decay. The use of porcelain as a filling-material gives to our patients fillings that harmonize with the natural teeth and enables us to perform æsthetic operations in such manner that the nervous strain is lessened upon both patient and dentist.

## Figures and Tables

**Fig. 1 f1:**
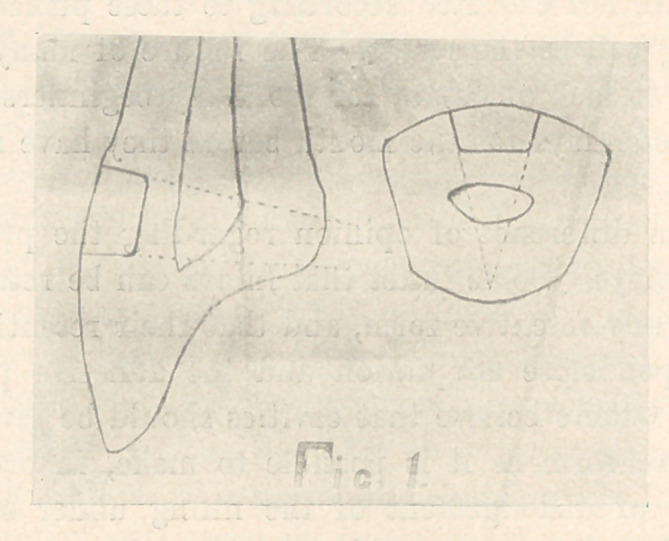


**Fig. 2 f2:**
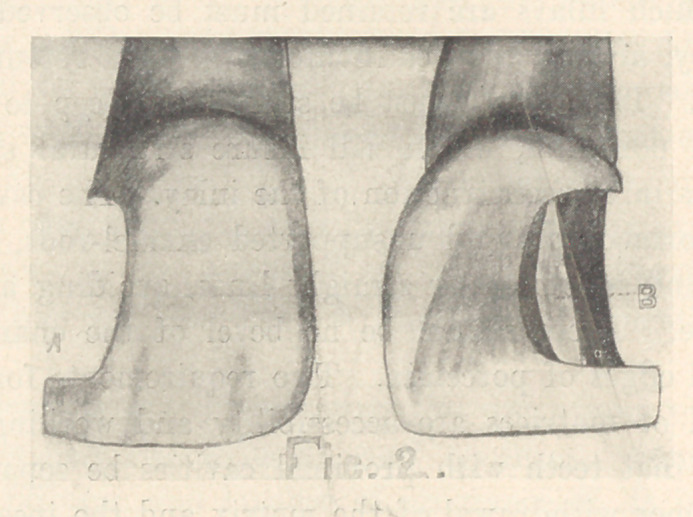


**Fig. 3 f3:**
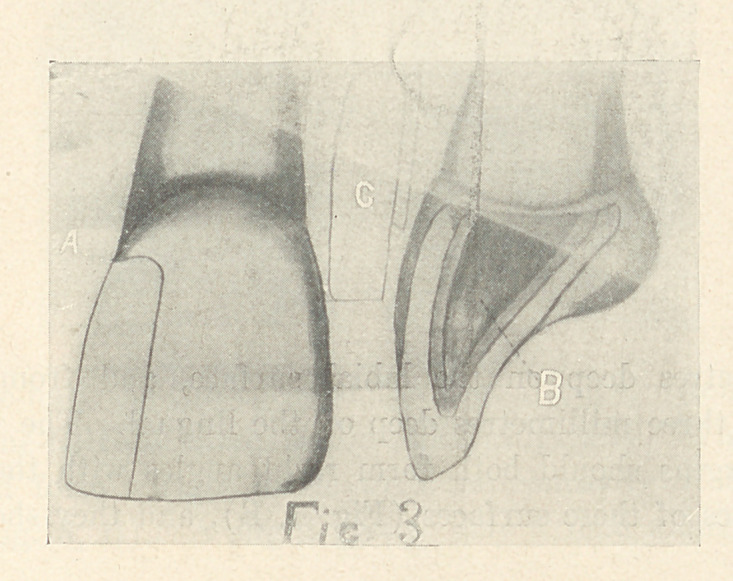


**Fig. 4 f4:**
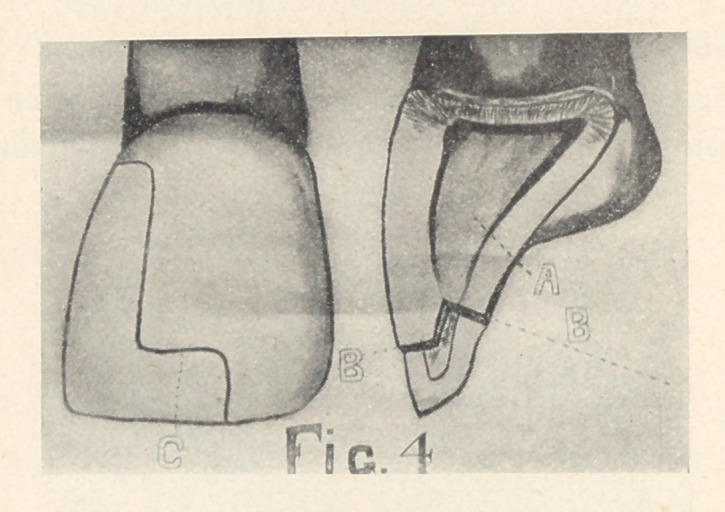


**Fig. 5 f5:**
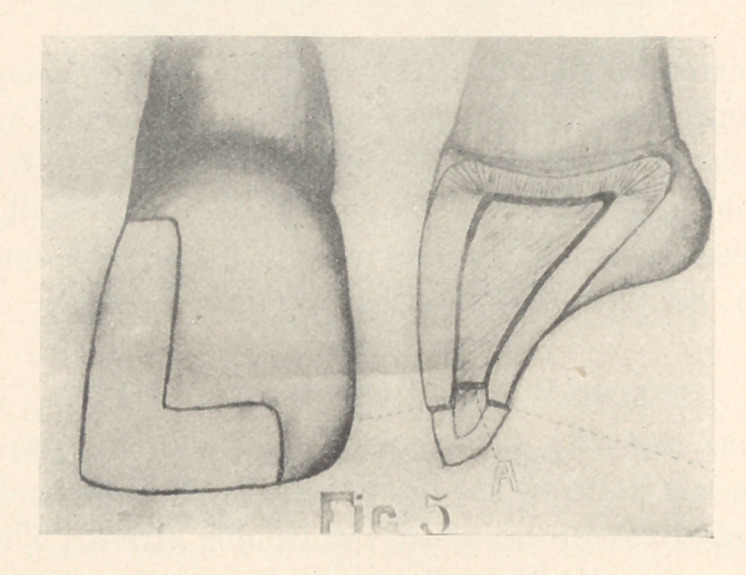


**Fig. 6 f6:**
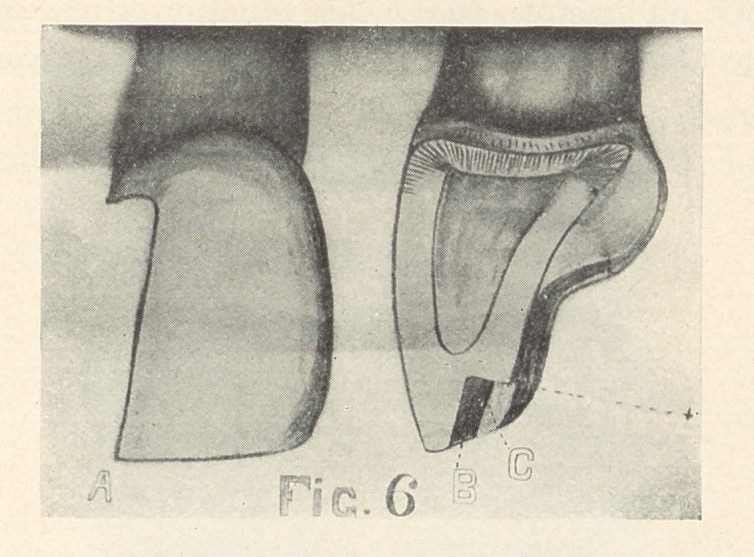


**Fig. 7 f7:**
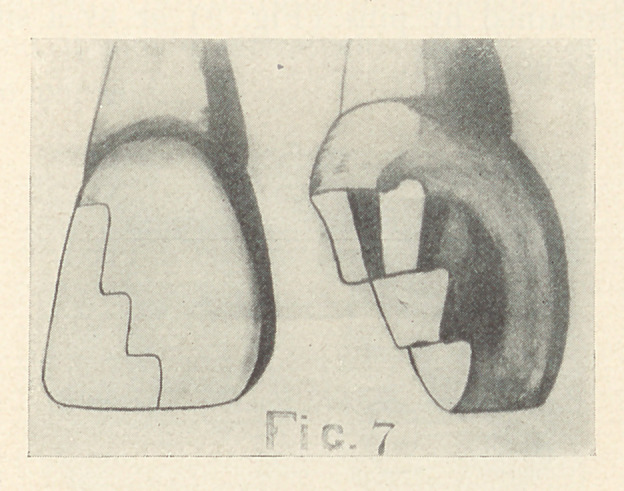


**Fig. 8 f8:**
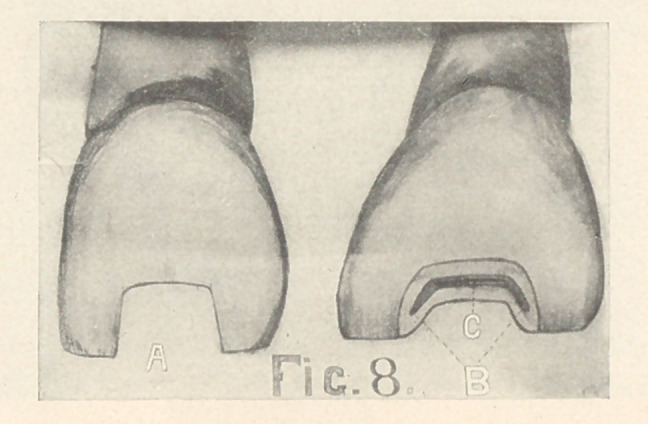


**Fig. 9 f9:**
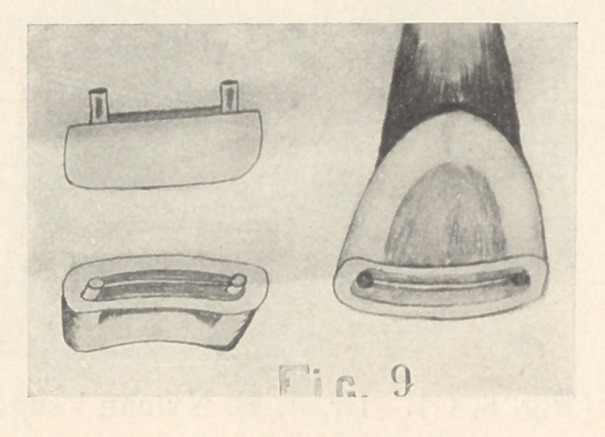


**Fig. 10 f10:**
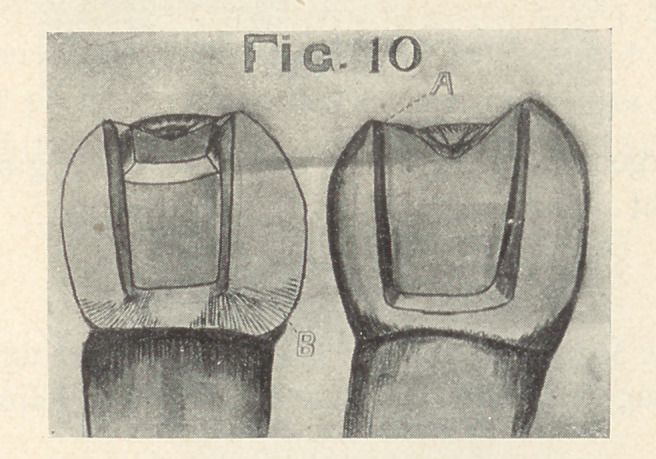


**Fig. 11 f11:**
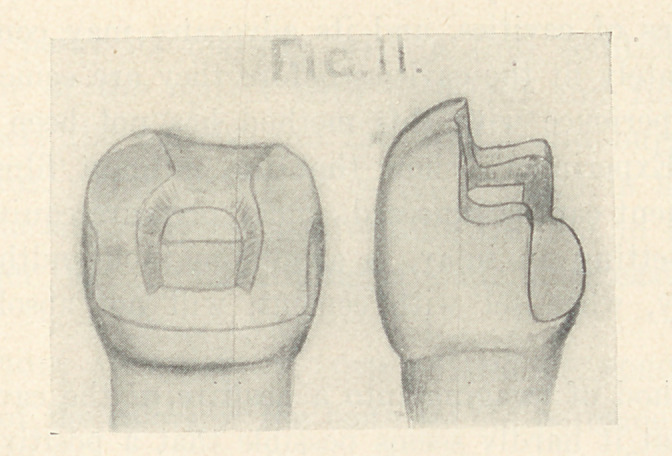


**Fig. 12 f12:**
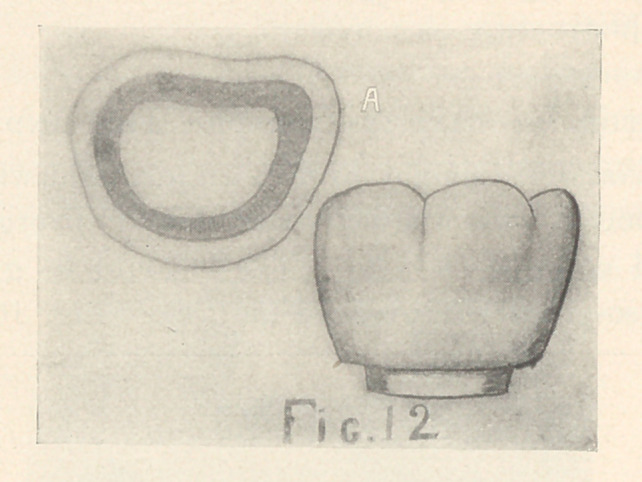


**Fig. 13 f13:**